# Influence of Manufacturing Parameters on Microstructure and Hydrogen Sorption Behavior of Electron Beam Melted Titanium Ti-6Al-4V Alloy

**DOI:** 10.3390/ma11050763

**Published:** 2018-05-10

**Authors:** Natalia Pushilina, Maxim Syrtanov, Egor Kashkarov, Tatyana Murashkina, Viktor Kudiiarov, Roman Laptev, Andrey Lider, Andrey Koptyug

**Affiliations:** 1 School of Nuclear Physics, National Research Tomsk Polytechnic University, Tomsk 634050, Russia; pushilina@tpu.ru (N.P.); maxim-syrtanov@mail.ru (M.S.); tatyanavolokitina@gmail.com (T.M.); victor31479@mail.ru (V.K.); laptevrs@tpu.ru (R.L.); lider@tpu.ru (A.L.); 2 Sports Tech Research Centre, Mid Sweden University, Akademigatan 1, SE-831 25 Östersund, Sweden; andrey.koptyug@miun.se

**Keywords:** electron beam melting, additive manufacturing, titanium Ti-6Al-4V alloy, hydrogen

## Abstract

Influence of manufacturing parameters (beam current from 13 to 17 mA, speed function 98 and 85) on microstructure and hydrogen sorption behavior of electron beam melted (EBM) Ti-6Al-4V parts was investigated. Optical and scanning electron microscopies as well as X-ray diffraction were used to investigate the microstructure and phase composition of EBM Ti-6Al-4V parts. The average α lath width decreases with the increase of the speed function at the fixed beam current (17 mA). Finer microstructure was formed at the beam current 17 mA and speed function 98. The hydrogenation of EBM Ti-6Al-4V parts was performed at the temperatures 500 and 650 °С at the constant pressure of 1 atm up to 0.3 wt %. The correlation between the microstructure and hydrogen sorption kinetics by EBM Ti-6Al-4V parts was demonstrated. Lower average hydrogen sorption rate at 500 °C was in the sample with coarser microstructure manufactured at the beam current 17 mA and speed function 85. The difference of hydrogen sorption kinetics between the manufactured samples at 650 °C was insignificant. The shape of the kinetics curves of hydrogen sorption indicates the phase transition α_H_ + β_H_→β_H_.

## 1. Introduction

Titanium and its alloys are widely used as structural materials mainly in aerospace industry due to low density (light weight), corrosion and fatigue resistance, high-temperature strength, fracture toughness, and low Young’s modulus [1,2,3]. Two-phase (α + β) titanium alloys are used to produce such critical and loaded parts as discs, working and guide blades, compressor rings, and other components. The operating temperatures of titanium alloys in aircraft engines vary from 120 to 580 °C [4,5]. During operation in aggressive environments containing hydrogen and oxygen at high temperatures, the physical and mechanical properties of titanium alloys significantly deteriorate. Hydrogen embrittlement is a serious problem for titanium alloy products because they are used in corrosive environments and are subjected to hydrogenation during operation [6,7,8,9,10]. Hydrogen absorbed by the products precipitates as a brittle hydride phase, leading to degradation of mechanical properties of titanium-based alloys. The kinetics and rate of hydrogen embrittlement of metals depends on many factors: type of crystal lattice, chemical composition, microstructure, defect state, temperature, the pressure and hydrogen concentration, and others. The study of various factors that influence hydrogen absorption by titanium alloys is important for the protection of alloys against hydrogen embrittlement and improvement of the mechanical properties by thermohydrogen processing [11,12]. The kinetic curves of hydrogen absorption and hydrogen desorption, the P–C isotherms [13,14], titanium alloy-hydrogen phase diagrams [13,15,16], and changes in microstructure under the influence of hydrogen are of special interest for investigation. The study of hydrogen absorption by metals and alloys becomes more important with introduction of new advanced technologies for manufacturing metal products. Additive manufacturing (AM) is actively introduced to the production of functional parts made from metal materials such as steel, aluminum, Ni-based superalloys, and titanium alloys [17,18,19,20]. Additive manufacturing technologies present a promising direction for manufacturing metal products directly from metal powder with minimal postprocessing [21,22,23]. The use of AM offers opportunities to speed up the manufacturing process, save metal, and produce lighter structures with complex shapes and geometries that cannot be achieved by traditional methods.

Electron beam melting (EBM) is an additive manufacturing method, where successive layers of metal powder are melted by high-power scanning electron beam [17,21]. The process takes place in high vacuum at elevated temperatures, which helps to significantly minimize thermally induced residual stresses. The structure and properties of the produced part depend on powder composition, thickness of the part, beam current, beam speed, scanning strategies (including line offset), energy input, and others. For example, in the Arcam EBM systems used in this study, the actual beam scanning speed can be controlled by the speed function (SF). Thus, the regularities of structure formation and evolution, which depend on the additive manufacturing parameters, are of great practical importance for creating products with a unique set of physical and mechanical properties. A large number of works have been devoted to investigating the influence of the electron beam melting parameters on the structure of titanium Ti-6Al-4V parts produced by EBM. It is known that the formation of the structural-phase state of Ti-6Al-4V alloy occurs as the result of powder melting at the temperature of 1900 °C and subsequent rapid cooling to the temperature of ~700 °C, followed by cooling to room temperature. If the titanium product is maintained above 700 °C in the EBM manufacturing process, a rather fine annealed α + β-structure has been observed [24]. The authors noted that the β-grains originated nonuniformly from the boundary layers either on the built plate or on the surfaces of the parts during the EBM process. They have been found to be formed from a partially molten powder in the surrounding layer on the surfaces of the parts. Safdar et al. [25] showed that the prior β phase, in the form of columnar grains, grows along the build direction and Widmanstätten α platelets are present in the structure of EBM Ti-6Al-4V alloy. The microstructure and porosity formed in Ti-6Al-4V samples produced by EBM over a range of melt scan speeds, from 100 mm/s to 1000 mm/s, were investigated in [26]. It has been shown that the increase in the melt scan rate during the EBM process of oriented Ti-6Al-4V cylinders reduces the cooling (solidification) rate, which leads to the decreasing α phase acicular grain width, as well as to the increase of proportion of α’-martensite plate. This refinement of microstructure improves the microhardness (HV) for the horizontal built cylinders. Correspondingly, with the increase of the melt scan rate due to the formation of unmelted powder volumes within the layers, the porosity increases. Al-Bermani [27] and Murr et al. [28] also showed the formation of martensitic phase in the samples during the EBM process. Juechter et al. [29] investigated the influence of scanning velocity on the EBM process and showed that the scanning speed up to 6.4 m/s^−1^ resulted in dense samples. Hrabe and Quinn demonstrated the effect of the distance from substrate, part size, energy input, orientation and location on the microstructure, and mechanical properties of Ti-6Al-4V fabricated by EBM [30]. Guo et al. studied the influence of beam current, scanning velocity, and scanning line in the range 2–18 mA, 250–2000 mm/s, and 2–50 mm, respectively. The authors found that there is α‘-martensite within the top region in samples of two types, which indicates that the primary β phase is first transformed into α‘-martensite and then decomposes into α/β phase [31]. The beam current and scanning velocity strongly influence the energy density and solidification rate. At the high-energy density, the liquid flows and spreads easier, filling in the pores and leading to a dense surface free of agglomerates. Tammas-Williams et al. [32] investigated the influence of the filling strategy on the formation of defects during EBM. It has been conclusively shown that the pores/defects are not randomly distributed, as strong correlation has been found with the process parameters and strategies used to outline (contouring) and infill (hatching) a part section. With the standard built parameters, it has been found that the vast majority of voids were small spherical gas pores. Wang [33] studied the impact of scanning velocity (speed function 20, 36, 50, and 65) on the microstructural variations and orientation. The authors found that the samples produced at SF50 have the highest Vickers hardness and elastic modulus, which results from its finest microstructure and the weakest texture. Antonysamy et al. investigated the effect of built geometry on the grain structure and texture in the EBM Ti-6Al-4V samples [24]. Thus, the structure evolution and properties of titanium alloys significantly depend on the manufacturing parameters. At the same time, the microstructure and phase composition make the main contribution to the metal–hydrogen interaction. The effect of hydrogen treatment on the structure and mechanical properties of the samples made from Ti-6Al-4V powder by selective laser melting method were reported in [11]. However, hydrogen sorption behavior of SLM Ti-6Al-4V has not been studied by the authors. Moreover, there is no data about hydrogen sorption by additively manufactured titanium alloys, nor about the influence of manufacturing parameters on hydrogen interaction with AM Ti-6Al-4V. The purpose of this work is to study the influence of manufacturing parameters of electron beam melting on microstructure and hydrogen sorption behavior of EBM Ti-6Al-4V parts.

## 2. Materials and Methods

### 2.1. Samples Preparation

Titanium Ti-6Al-4V parts produced by the method of electron beam melting were investigated in this work. The parts were produced with ARCAM A2 EBM (Arcam AB, Mölndal, Sweden) machine using powder of titanium Ti-6Al-4V alloy (Ti6Al4V ELI) [34]. The powder was purchased from Arcam AB and has an average grain size distribution from 50 to 150 μm. The samples were coin-shaped with the diameter 8 mm and height 2 mm. All used samples were manufactured in the same batch with the build direction parallel to the round surface. Powder layer thickness was 70 μm. The manufacturing parameters are shown in Table 1. All the samples were carefully blasted in the Arcam powder recovery system using the same precursor powder. Then, all the samples were mechanically grinded to obtain homogeneous surface.

### 2.2. Experimental Procedure

The gas-phase hydrogenation of the samples was performed using gas reaction controller (AMC, Pittsburgh, Pennsylvania, PA, USA) equipment at the temperatures 500–650 °С at constant pressure 1 atm. The special software (AMC, Pittsburgh, Pennsylvania, PA, USA) for the gas reaction controller equipment was used to control and analyze the process of hydrogen sorption and reveal the specifics of hydrogen interaction with materials [35]. After hydrogenation, the samples were incubated in an inert gas atmosphere at the temperature of 650 °C and pressure 2 atm for 2 h in order to achieve uniform distribution of hydrogen in the volume. The heating and cooling rates were 6 °С/s and 1 °С/s, respectively. The hydrogen concentration in the samples was measured by the method of melting in inert gas media (argon) using a hydrogen analyzer RHEN602 (LECO, Saint Joseph, Michigan, MI, USA). Hydrogen concentration in the samples before hydrogenation was 0.008 wt %. The measured hydrogen concentrations in the samples after hydrogenation were 0.3 wt %.

Microstructure of the samples was analyzed by optical microscopy (OM) using AXIOVERT-200MAT (Zeiss, Göttingen, Germany) in the center of collective usage “Nanotech”, ISPMS SB RAS. Additionally, the detailed microstructure analysis was performed by scanning electron microscopy using S-4800 (Hitachi, Tokyo, Japan). The samples were etched out by Kroll’s reagent (2 mL HF, 6 mL HNO_3_ and 92 mL H_2_O) to reveal the structure of the samples after mechanical polishing. The phase identification and structural investigations were performed by X-ray diffraction (XRD). X-ray diffraction studies were performed with CuKα radiation (1.5410 Å wavelength) using XRD-7000S diffractometer (Shimadzu, Kyoto, Japan) in Bragg-Brentano geometry from 30° to 80° with the scan speed of 10.0°/min, the sampling pitch of 0.0143°, and the preset time of 42.972 s at 40 kV and 30 mA. The diffraction patterns were collected using OneSight wide-range array high speed detector with 1280 channels.

## 3. Results

### 3.1. Microstructure of EBM Ti-6Al-4V Samples

**Figure 1:**
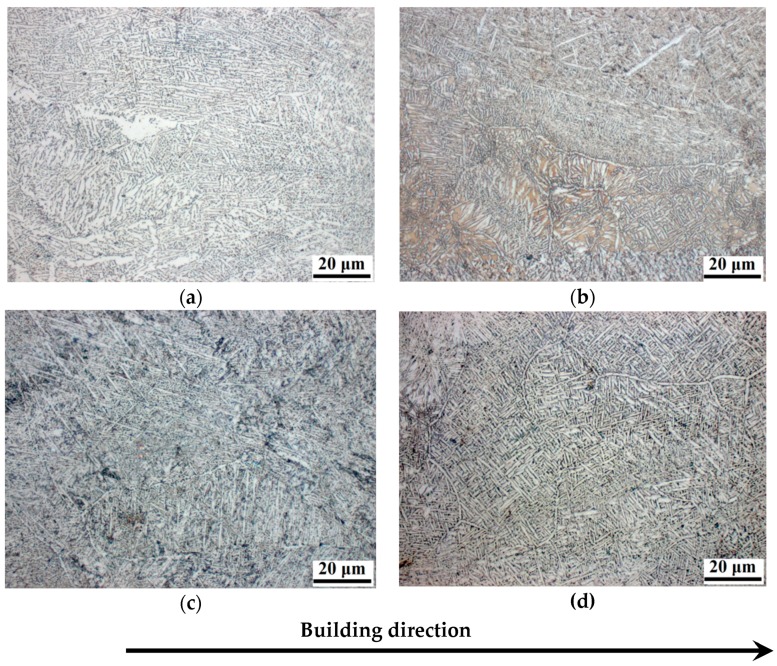
Optical microscopy images of microstructures of sample: (**a**) S1; (**b**) S2; (**c**) S3; (**d**) S4.

Figure 1 shows the optical images of the samples structure. A lamellar microstructure with fine α laths, which formed as a result of rapid cooling from the high-temperature β phase, is observed in all the EBM Ti-6Al-4V samples. The microstructure is characterized by the presence of relatively large prior β-grains (width varies from 40 to 100 μm). The internal volume of β-grains is separated by α-plates collected in colonies. The formation of colonies is attributed to the fact that the β→α transformation begins independently in several sections of the prior β phase grains.

**Figure 2:**
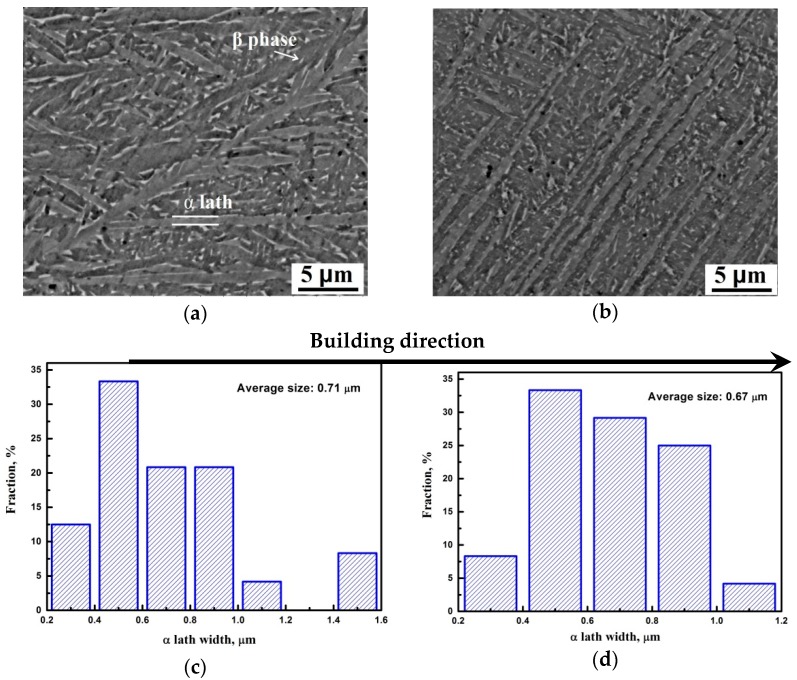
SEM images of the samples (**a**,**b**) and histogram (**c**,**d**) of the thickness distribution of α plates, respectively: (**a**,**c**)—S1; (**b**,**d**)—S2.

**Figure 3:**
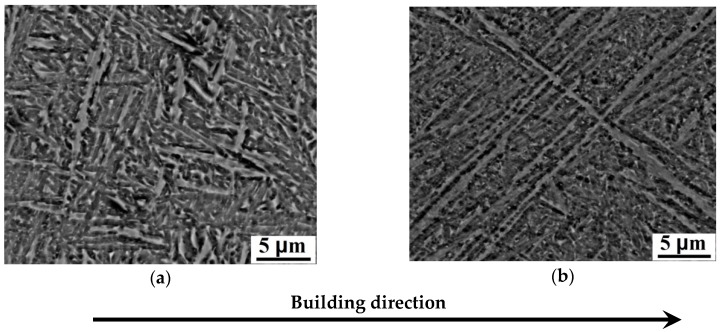

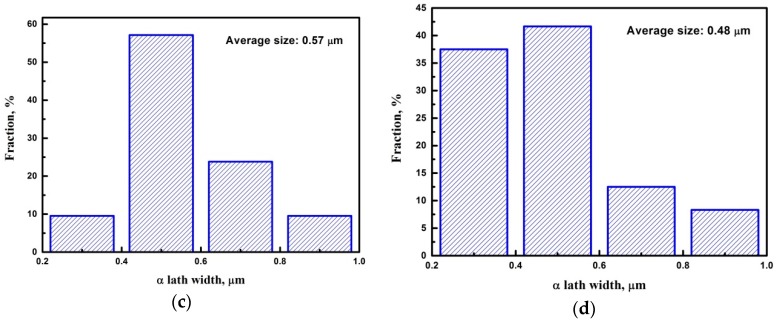
SEM images of the samples (**a**,**b**) and histogram (**c**,**d**) of the thickness distribution of α plates, respectively: (**a**,**c**)—S3, (**b**,**d**)—S4.

The detailed structure analysis along with the distribution of α lath width, obtained using scanning electron microscopy, is shown in Figure 2. The body-centered cubic structure of β phase in EBM-built Ti-6Al-4V formed as discrete flat rods embedded in the continuous α phase with hexagonal close-packed structure (Figure 2). The thicker α lath is observed in the samples prepared at beam current 17 mA and speed function 85 (Figure 2a). In this case, besides the width of α laths in the structure is mainly 0.4–0.6 μm, larger α plates with the width of 1.4–1.6 μm are also observed (Figure 2a,c). The thinner α plates are observed in the sample prepared at the beam current 17 mA and speed function 98 (Figure 3b,d). The structure of this sample comprises plates predominantly with a width of 0.2–0.5 μm (Figure 3b,d). It was found that the average size of α plates decreases with increasing speed function with a constant value of beam current (17 mA) (Figure 2a–c). Reduction of the beam current from 17 to 13 mA at the fixed SF85 leads to the decrease in the dimensions of the α plates, but this effect is less noticeable in comparison with the effect of the increase of the speed function.

**Figure 4:**
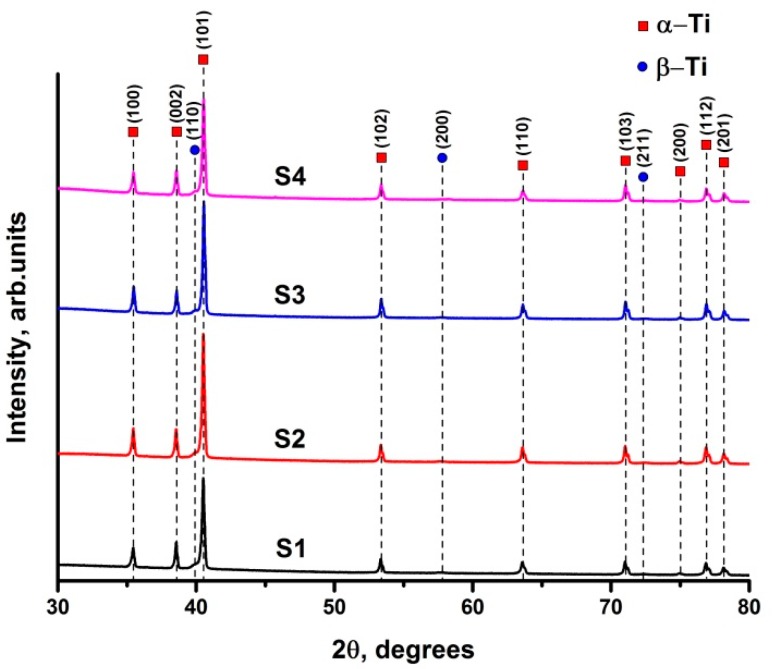
Diffraction patterns of Ti-6Al-4V samples produced by electron beam melting at different manufacturing parameters.

Figure 4 shows the results of X-ray diffraction analysis of electron beam melted Ti-6Al-4V parts at different manufacturing parameters (see Table 1). The phase content and lattice parameters are presented in Table 2. According to XRD analysis, the α-Ti (α′-Ti) phase with hexagonal close-packed (hcp) lattice and β-Ti phase with body-centered cubic (bcc) lattice were observed in all the samples. It is impossible to perceive the differences between α-Ti and α′-Ti in the EBM samples using XRD [35,36]. The change in electron beam melting parameters does not significantly affect the structure and phase composition of the additively manufactured Ti-6Al-4V samples. The content of β phase in the samples varies from 2.4 to 3.1 vol %. The accuracy of XRD analysis does not allow revealing of the peculiarities of the EBM parameters’ effect on the formed β titanium phase content. It is assumed that within the indicated range, the change in the parameters does not significantly influence the phase composition of the EBM samples. It is known that the α-Ti to β-Ti ratio can be controlled by different heat treatment temperatures and cooling rates [37]. In this investigation, the cooling rates of the electron beam melted samples were the same.

### 3.2. Hydrogen Sorption

**Figure 5:**
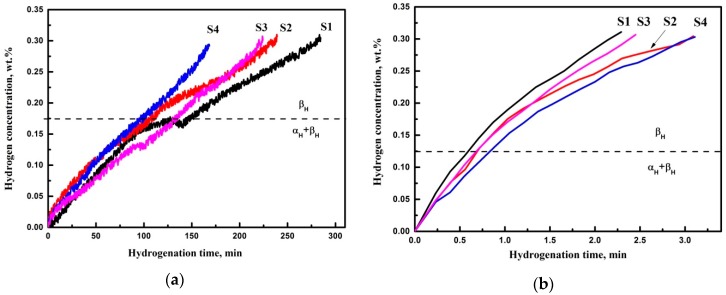
The kinetic curves of hydrogen absorption: (**a**) T = 500 °С; (**b**) T = 650 °С.

Figure 5a,b show the hydrogen sorption curves by the samples at temperatures of 500 and 650 °C, respectively. The intensity of the hydrogen sorption process is characterized by the angle of inclination of the kinetic curves “hydrogen concentration–hydrogenation time”. There are changes in the slope on the sorption curves at 500 °C that were caused by the change of hydrogen diffusion rate in the material due to phase transitions. It is possible to determine the boundaries of single-phase and two-phase regions of the system by changing the slope of the curves [13]. Thus, the changes in slope and plateaus on the kinetic curves of hydrogen sorption are associated with phase transitions. This transition occurs as follows: α_H_ +β_H_→β_H_, which in accordance with the (Ti-6Al-4V)-H phase diagram and literature data under the experimental conditions [13]. Thus, the formation of hydrogen solid solution in the α and β phases occurs at the initial stage. Then, the transition to β phase occurs when a certain concentration is reached.

The kinetic curves of hydrogen absorption at the temperature of 500 °C for the samples produced under different parameters differ significantly. Under the experimental conditions, sample S4 manufactured at the beam current 17 mA and SF 98 absorbed hydrogen most intensely. The specified hydrogen concentration of 0.3 wt % was achieved in 170 min, whereas it took 270 min in sample S1 produced with the beam current 17 mA and SF 85. There is the correlation between the sorption rate and the microstructure of the EBM Ti-6Al-4V samples. Reduction of the α plate size leads to the increase in the average hydrogen sorption rate.

The increase in the hydrogenation temperature to 650 °C leads to the significant increase in the rate of hydrogen absorption by the samples (Figure 5b). In this case, the difference in the rate of hydrogen absorption by the samples manufactured under different parameters practically disappears.

## 4. Discussion

The formation of the microstructure and the phase state of Ti-6Al-4V alloy occurs as the result of powder melting at the temperature of 1900 °C and subsequent rapid cooling to the temperature of ~700 °C followed by cooling to room temperature [24]. At the same time, Antonysamy [24] noted that the growth rate, temperature gradient, melt pool shape, travel speed, undercooling, and alloy constitution will all control the final microstructure of a solidifying melt pool in AM. The rapid cooling of the lamellar structure below the β transus results in the formation of finer laths and smaller α colonies, whereas slow cooling results in thick α laths and coarse α colonies, typically observed in castings [38,39]. The increase in the speed function from 85 to 98 with the fixed beam current (17 mA) leads to the decrease in the average size of α plates according to the microstructure studies carried out and discussed in this paper. It has been established in [40] that the molten pool length was found to decrease with the speed function index increase; the width appeared to decrease with increasing speed function. Thus, the structure refinement performed within the present work with the increase of the speed function is due to the decrease of the molten pool length, respectively. Meanwhile, the smaller the melt pool width, the greater the cooling rate. It has been established in [41] that the highest beam current results in the largest molten pool size. Thus, the reduction of the beam current from 17 to 13 mA with the fixed SF causes the decrease of the size of the α plates due to the lower molten pool size and, correspondingly, higher cooling rate. 

The structure formed as the result of electron beam melting has a significant effect on the kinetics of hydrogen absorption by the samples (Figure 5a). The kinetics of hydrogen absorption by titanium alloys is significantly affected by the grain size and shape and the fraction and distribution of the β phase. Samples with fine grains absorb hydrogen more intensely than samples with large grains [42]. Titanium having a structure consisting of elongated grains absorbs a predetermined amount of hydrogen several times faster than titanium having an equiaxed structure [42,43]. Tal-Gutelmacher [44] reported that the absorbed hydrogen concentration in the fully lamellar alloy is always higher than in the duplex microstructure, irrespective of the hydrogen charging conditions. It has been shown that the diffusion of hydrogen along grain boundaries (GBs) occurs much faster (by four orders of magnitude) compared to α phase [45]. Gaddam et al. [46] supposed lower hydrogen diffusivity through EBM Ti-6Al-4V with smaller α lamellar colonies and less continuous β phase compared to cast Ti-6Al-4V with long continuous β phase. However, the effect of GBs (including boundaries between α platelets in colonies) and β phase distribution on H diffusion in ultrafine microstructures has not been revealed. In the current work, average hydrogen sorption rate is compared among EBM Ti-6Al-4V alloy samples with different microstructures. The samples with finer microstructure demonstrate higher average hydrogen sorption rate due to the decreased size of α plates and the corresponding distribution of β phase (increased number of β rods between the α plates). Moreover, there is significant difference in the hydrogen sorption behavior (Figure 5a), which could be affected by several factors: number of GBs, β phase content, and distribution as well as size of α laths. In the α+β titanium alloys, the amount of β phase significantly affected the diffusion of hydrogen, even at low β phase content. The diffusion coefficient of hydrogen in different titanium phases as the function of temperature was reported in [47,48]:

(1) $$D_{\alpha }=3\times 10^{-6}\exp\left[\frac{-14700\pm 650}{RT}\right]$$

(2) $$D_{\beta }=1.95\times 10^{-7}\exp\left[\frac{-6640\pm 500}{RT}\right]$$

where D is the diffusion coefficient (m^2^/s), R is the gas constant (cal mol^−1^ K^−1^), T is the temperature (K). It has been noted that the diffusion of hydrogen in Ti-6Al-4V significantly depends on microstructure and β phase content and could vary from 10^−13^ to 10^−10^ m^2^/s at 20 °C [49]. According to these equations, the calculated values of H hydrogen diffusion in titanium at 500 °C (773 K) are D_α_ = 2.1 × 10^−10^ m^2^/s and D_β_ = 2.6 × 10^−9^ m^2^/s. Thus, hydrogen preferentially diffuses through the β phase and interacts with α phase along the α/β boundaries. Nevertheless, we suppose that the hydrogen also has a high diffusion rate not only for the GBs of primary β phase, but also between the boundaries of α plates, which mainly consist of β phase rods. The visible change of hydrogenation behavior (the curves shape demonstrated in Figure 4) indicates different sorption processes. At the initial stage, hydrogen mainly diffuses through the β phase and GBs, with α phase reaching the saturation concentration of α_H_+β_H_ phases. Then, the transition of α_H_ + β_H_→β_H_ accompanied with the increase in the hydrogen absorption rate (after plateau in Figure 5) is observed. A similar observation was reported in [14]. It is supposed that the difference between the hydrogen concentration of phase transition α_H_ + β_H_→β_H_ is caused by β phase content and defect structure of the material, while the dimensions of α phase plates and β phase distribution are caused by the width of phase transition. In other words, smaller α plates and developed β boundaries (β rods) between the α plates can promote fast diffusion of hydrogen and saturation of individual α plates. Thus, the width of phase transition is higher in the sample with coarse microstructure (1) and negligible in the sample with fine microstructure (4). With the temperature increase to 650 °С, change in the behavior of kinetic curves of hydrogen absorption can be explained by significant increase of the diffusion rate in α phase (D_α_ = 1 × 10^−9^ m^2^/s), while the diffusion rate in β phase comprises D_β_ = 5.3 × 10^−9^ m^2^/s. The change in the hydrogen sorption behavior with increasing hydrogenation temperature to 650 °С is attributed to enhanced hydrogen diffusion in α phase (D_α_ = 1 × 10^−9^ m^2^/s), while the diffusion in the β phase is D_β_ = 5.3 × 10^−9^ m^2^/s. The increase of the temperature to 650 °C reduces the transformation time of α_H_ + β_H_→β_H_, which is confirmed by disappearance of the plateau on the kinetic curves (Figure 5b). Moreover, the given concentration of hydrogen is achieved in a short time (2–3 min.). Thus, at the temperatures of 650 °C and above, the difference in the microstructure of the samples does not significantly affect the kinetics of hydrogen absorption.

## 5. Conclusions

The influence of manufacturing parameters (beam current and speed function) on microstructure, phase composition, and hydrogen sorption kinetics of the Ti-6Al-4V parts produced by electron beam melting has been investigated. The following points have been highlighted:

1. The average α lath width decreases with the increase of the speed function at the fixed beam current (17 mA). At the fixed speed function, the decrease of the α lath width also occurs when changing the beam current from 17 to 13 mA. Finer microstructure has been formed at the beam current (BC) 17 mA and speed function 98, while coarser microstructure has been formed at 17 mA and 85, respectively. The bcc β phase in EBM Ti-6Al-4V has been formed as discrete flat rods embedded in the continuous hcp α phase. The phase composition of the samples changes insignificantly at the varied parameters. The content of β phase varies from 2.4 to 3.1 vol %.
2. Microstructure has significantly affected hydrogen sorption kinetics of EBM Ti-6Al-4V parts during the gas-phase hydrogenation at 500 °C. The average hydrogen sorption rate was higher in the sample manufactured at BC 17 mA and SF 98 due to finer microstructure (finer α lath) and distribution of β phase. Lower hydrogen sorption was demonstrated in the sample with BC 17 mA and SF 85. The shape of the kinetics curves indicates the phase transition α_H_ + β_H_→β_H_, which depends on the dimensions of α plates and β phase content and distribution.
3. Hydrogen sorption kinetics at 650 °C has not significantly changed at the indicated manufacturing parameters due to the increase of hydrogen diffusion in α phase. Thus, the transition α_H_ + β_H_→β_H_ proceeds rapidly as compared to hydrogenation at 500 °C. The hydrogenation time to 0.3 wt % is about 2–3 min.

## Figures and Tables

**Table 1:** Electron beam melting parameters.

Sample	Beam Current (BC), mA	Speed Function (SF)	Beam Speed, mm/s
S1	17	85	3227.7
S2	15	85	2797.6
S3	13	85	2797.6
S4	17	98	3218.8

**Table 2:** Phase composition and lattice parameters in the electron beam melting (EBM) samples.

Sample	Phase	Phase Content, vol %	Lattice Parameters, Å	c/a
S1	Ti_hexagonal	96.9	A = 2.9253 c = 4.6709	1.597
	Ti_cubic	3.1	A = 3.1954	-
S2	Ti_hexagonal	97.1	A = 2.9248 c = 4.6717	1.597
	Ti_cubic	2.9	A = 3.1905	-
S3	Ti_hexagonal	97.6	a = 2.9253 c = 4.6720	1.597
	Ti_cubic	2.4	a = 3.1926	-
S4	Ti_hexagonal	97.1	a = 2.9245 c = 4.6710	1.597
	Ti_cubic	2.9	a = 3.1911	-
